# A novel immunohistochemical method for estimating cell cycle phase distribution in ovarian serous neoplasms: implications for the histopathological assessment of paraffin-embedded specimens

**DOI:** 10.1038/sj.bjc.6601660

**Published:** 2004-03-30

**Authors:** I S Scott, T M Heath, L S Morris, S M Rushbrook, K Bird, S L Vowler, M J Arends, N Coleman

**Affiliations:** 1 MRC Cancer Cell Unit, Hutchison/MRC Research Centre, Hills Road, Cambridge CB2 2XZ, UK; 2 Centre for Applied Medical Statistics, University Forvie Site, Robinson Way, Cambridge CB2 2SR, UK; 3 Addenbrooke's Hospital, Department of Molecular Histopathology, University of Cambridge, Hills Road, Cambridge CB2 2QQ, UK

**Keywords:** ovary, serous cystadenocarcinoma, borderline tumours, minichromosome maintenance proteins, cell cycle

## Abstract

We have investigated whether immunohistochemical markers can identify differences in cell cycle phase distribution in ovarian serous neoplasms, including borderline tumours of different grades. Sections of normal ovary (*n*=18), serous cystadenoma (*n*=21), borderline serous tumours (*n*=21) and serous cystadenocarcinoma (*n*=15) were analysed by immunohistochemistry using markers of cell cycle entry (Mcm-2) and cell cycle phase, including cyclin D1 (mid-to-late G1), cyclin A (S phase), cyclin B1 (G2 phase) and phosphohistone H3 (mitosis). Double-labelling confocal microscopy confirmed marker phase specificity and phase estimations were corroborated by flow cytometry. On progression from normal ovary through serous cystadenoma and borderline tumours to cystadenocarcinomas, expression of Mcm-2 (*P*<0.0001), cyclin D1 (*P*=0.002), cyclin A (*P*<0.0001), cyclin B1 (*P*<0.0001) and phosphohistone H3 (*P*<0.0001) increased, paralleled by an increase in the S-phase fraction (cyclin A : Mcm-2 ratio; *P*=0.002). Borderline tumours of increasing grade also showed increased Mcm-2 and cyclin A expression, together with an increase in the S-phase fraction. Immunohistochemistry can be used to estimate cell cycle phase distribution in ovarian serous neoplasms, giving results similar to flow cytometric analysis and enabling direct assessment of tumour heterogeneity. Immunohistochemical estimates of the S-phase fraction may identify serous borderline tumours likely to exhibit malignant progression and/or select serous cystadenocarcinomas likely to respond to adjuvant therapy.

Neoplasms of the ovarian surface epithelium account for 60% of all ovarian tumours. They are divided into serous, mucinous, endometrioid and other types, of which serous are the most common (Fox and Buckley, 1992; Zaloudek, 2000). Serous tumours are classified further into benign, borderline and malignant neoplasms. The borderline group is of special interest because, although the majority of these tumours will behave in a benign manner, 15–30% will progress to an invasive malignancy and such tumours have a poor prognosis. The ability to differentiate reliably between those borderline tumours that will behave in a benign manner and those with a propensity to progress is essential, as tumours described as borderline on histological examination are treated differently from those displaying a malignant phenotype (Russell, 1994; Dietel and Hauptmann, 2000). Indeed, in the younger patient, the diagnosis of a borderline tumour may result in conservative treatment, and failure to remove the contralateral ovary. As recurrences in borderline tumours are often bilateral, conservative treatment and the failure to remove both ovaries at initial surgery often results in aggressive disease with a very poor prognosis (Russell, 1994). At the present time, there is no reliable method of histological differentiation between those borderline tumours destined for malignant progression and those that are likely to behave in a benign manner. Consequently, some tumours that will progress to a malignant phenotype will be missed despite careful histological examination (Dietel and Hauptmann, 2000).

It has been suggested that ovarian tumours having a high S-phase fraction behave in a more aggressive manner (Skirnisdottir *et al*, 2001) and that, conversely, tumours with a low proliferation index respond poorly to chemotherapy (Itamochi *et al*, 2002). We have developed a strategy, based on immunohistochemistry, to allow cell cycle phase analysis to be performed on paraffin-embedded tissue in the routine diagnostic laboratory (Scott *et al*, 2003). Such an approach will be applicable in any histopathology laboratory capable of performing immunohistochemistry, unlike the complex techniques of flow cytometry and cytogenetics, which are difficult to apply in the diagnostic setting. We ultimately aim to determine whether or not cell cycle phase parameters, as determined by immunohistochemistry, are of prognostic significance in ovarian tumour pathology, either in the prediction of outcome/rate of progression of ovarian serous cystadenocarcinomas or in the differentiation between low- and high-risk borderline serous tumours.

Minichromosome maintenance (MCM) protein 2 is one of six MCM proteins (MCM 2–7) that assemble in the prereplication complex and are essential for DNA replication in eukaryotic cells (Tye, 1999; Lei and Tye, 2001). All six proteins are abundant throughout the cell cycle and are broken down rapidly on differentiation or more slowly in quiescence (Musahl *et al*, 1998; Todorov *et al*, 1998). Antibodies against MCMs such as Mcm-2 or Mcm-5 have been shown to be of value in identifying malignant or premalignant lesions in a range of specimens (Williams *et al*, 1998; Freeman *et al*, 1999; Meng *et al*, 2001; Wharton *et al*, 2001; Davies *et al*, 2002; Going *et al*, 2002; Hunt *et al*, 2002). We have also demonstrated the potential for antibodies raised against cyclins D1, A, B1 and phosphorylated histone H3 to estimate cell cycle phase distribution by immunohistochemistry (Scott *et al*, 2003). Cyclin D1 is maximally expressed in mid-to-late G1 and is involved in the G1/S transition (Coverley *et al*, 2002). Cyclin A is generally maximally expressed in S phase, with only low-level expression in G2 (Pines and Hunter, 1992; Hunter and Pines, 1994; Yam *et al*, 2002; Scott *et al*, 2003). The exact pattern of expression appears, however, to vary according to the nature and type of cell line used, with some cell lines showing low-level cyclin A expression into early metaphase (Yam *et al*, 2002). Cyclin B1 is expressed as a cytoplasmic molecule in G2 but becomes located within the nucleus in early mitosis (M) until the breakdown of the nuclear membrane, at which point staining becomes diffuse (Pines and Hunter, 1992; Nasmyth, 1996). Phosphohistone H3 is specific for mitosis and is rapidly degraded on entry into G1 (Shibata and Ajiro, 1993). Antibodies against this set of markers might enable, therefore, *in situ* labelling of cells at all phases of the cell cycle, with the exception of early G1, for which no useful marker is currently available.

As this method of analysing cell cycle kinetics in the ovary is novel, we compared our findings with those obtained using the established method of flow cytometry. The relative phase specificity of each marker was also established in the ovary by double labelling and confocal microscopy to ensure that there was no significant coexpression of putative markers of adjacent cell cycle phases. Such data would be consistent with our observations in tumours at other anatomical sites (Scott *et al*, 2003). The ability to estimate the S-phase fraction in paraffin-embedded ovarian tumour tissue would allow this parameter to be included in existing diagnostic algorithms and may allow the identification of tumours that respond well to adjuvant therapy. The method would also enable retrospective studies to be performed on archival material, thus avoiding the need for lengthy prospective studies and the requirement for frozen material for reliable flow cytometric analysis.

## MATERIALS AND METHODS

### Resection specimens

Archival blocks of anonymised formalin-fixed, paraffin-embedded specimens of human ovary were obtained in accordance with Local Research Ethics Committee guidelines. The tissues examined represented normal ovary (*n*=18), serous cystadenomas (*n*=21), borderline serous neoplasms (*n*=21) and serous cystadenocarcinomas (*n*=15).

### Immunohistochemical staining of paraffin-embedded sections

Sections (5 *μ*m) were cut onto aminopropyltriethoxysilane (APES)-coated slides and were processed for immunohistochemistry as described previously (Freeman *et al*, 1999). Mouse monoclonal antibodies raised against the following antigens were used: minichromosome maintenance protein-2 (Mcm-2) (Davies *et al*, 2002); Cyclin D1 (Nova Castra, Newcastle, UK); Cyclin A (Nova Castra, Newcastle, UK); and Cyclin B1 (DAKO, Ely, UK). We also used rabbit polyclonal antibodies raised against Mcm-5 (Williams *et al*, 1998) to facilitate double labelling, and a rabbit polyclonal against phosphohistone H3 (Upstate Biotechnology, Lake Placid, NY, USA). Antigen retrieval was achieved by pressure cooking for 3 min in citrate buffer (pH 6.0), except for the cyclin D1 preparations for which antigen retrieval was achieved by heating in a programmable microwave oven (MicroMED T/T Mega, Sorisole, Italy) for 30 min at 98°C. We found that the staining pattern obtained with cyclin D1 was highly dependent upon the conditions used to facilitate antigen retrieval. A high pH buffer (DAKO high pH antigen retrieval solution (pH 9.6), DAKO, Ely, UK) and heating in a microwave oven produced predominantly nuclear staining, whereas neutral or low pH buffers, particularly when combined with pressure cooking, gave a mixed cytoplasmic/nuclear pattern. In addition, minimal background staining was achieved when cyclin D1 primary antibodies were detected using the EnVision system (DAKO, Ely, UK). Negative controls were performed by omitting the primary antibody. Sections of cervix showing various grades of intraepithelial neoplasia were used as positive controls.

### Double labelling studies

In order to test the validity of using antibodies to estimate cell cycle phase *in situ*, double labelling experiments were performed in ovarian serous borderline tumours and serous cystadenocarcinomas (*n*=6 in each group) as previously described (Scott *et al*, 2003). In the first series of reactions, Mcm-2 or Mcm-5 was combined with each of the four putative phase markers, cyclin D1, cyclin A, cyclin B1 and phosphohistone H3. We tested the hypothesis that if antibodies against MCMs identify all cells in cycle, none of the cyclins or phosphohistone H3 antibodies should detect cells negative for Mcm-2/5. The second series of reactions was designed to investigate the frequency of coexpression of the different markers of cell cycle phase. Markers were paired as follows: phosphohistone H3-cyclin D1 (putative markers of M and G1 phases); cyclin D1-cyclin A (G1 and S phases); cyclin A-cyclin B1 (S and G2 phases); and cyclin B1-phosphohistone H3 (G2 and M phases).

Where the primary antibodies had been raised in different species they were added together and incubated overnight. Following washing, both secondary antibodies (Alexa Fluro goat anti-mouse 488 and Alexa Fluro goat anti-rabbit 546; Molecular Probes) were added together and incubated for one hour. After further washing, slides were counterstained using 4,6-diamidino-2-phenylindole (DAPI) (Sigma, Poole, Dorset, UK), washed and mounted in fluorescent mounting medium (DAKO, Ely, UK).

When the primary antibodies were both mouse monoclonals a different procedure was performed. Initially, one of the primary antibodies was applied alone. After washing, the sections were incubated with Alexa Fluro goat anti-mouse 488 (Molecular Probes) and this was followed by a blocking step with F(ab)_2_ goat anti-mouse IgG fragments (Jackson Immuno Research Laboratories). A further washing step was then performed before incubation with the second primary antibody. Following a final washing step and incubation in Alexa Fluro goat anti-mouse 633 (Molecular Probes), the slides were counterstained and mounted as described above.

Images were viewed and assessed using a Zeiss Axioplan 2 confocal microscope at wavelengths of 488, 546 and 633 nm.

### Flow cytometry

A subset of serous cystadenocarcinomas (*n*=10) was selected for additional flow cytometric analysis, to allow comparison between the cell cycle phase distributions determined by flow cytometry and those estimated by immunohistochemistry. The cases were selected on the basis that the biopsy was composed almost entirely of tumour and contained a minimal inflammatory cell infiltrate, thus minimising contamination of the preparation by non-neoplastic cell nuclei.

Sections (50 *μ*m) were cut from paraffin blocks into Eppendorf tubes. The sections were dewaxed in xylene and taken through decreasing concentrations of alcohol until final equilibration was achieved in phosphate-buffered saline (pH 7.4). The samples were then incubated for 1 h at 37°C in the presence of 1 mg/ml collagenase and 1 mg/ml trypsin. The sample was then passed through a 23-G needle to disperse clumps and the nuclei were precipitated by centrifugation at 300 **g** for 5 min. RNA was removed by incubation in RNase A (100*μ*g ml^−1^) for 30 min at 37°C. The nuclei were then repelleted at 300 g for 5 min, washed in PBS and passed through a 70 *μ*m sieve. The nuclei were stained using propidium iodide (50 *μ*g ml^−1^) and were assessed using an argon laser tuned to 488 nm. The fraction of cells in each phase of the cycle was analysed using CELLQuest Software (Becton Dickinson, Cowley, UK).

### Quantitation of immunohistochemical staining

For sections of normal ovary and serous cystadenomas, the lining of serous inclusions and strips of cyst wall, respectively, were examined. The number of cells expressing each putative phase marker was determined both as a percentage of the total number of cells (termed a labelling index, LI) and as a percentage of the number of Mcm-2 positive epithelial cells (termed a labelling fraction, LF). LFs were determined to indicate the proportions of the cells in cycle that were in each cycle phase. For the borderline serous tumours and the serous cystadenocarcinomas, similar counts were performed, although for these tumours the percentage of the total number of cells expressing each antigen was established for three random high magnification microscopic fields rather than for epithelial strips. Approximately 1000 cells were counted for each case by three individual observers and the interobserver variation was less than 5%.

Cell cycle analyses obtained by immunohistochemistry were compared directly with those obtained by flow cytometry for 10 cases of serous cystadenocarcinoma. In the immunohistochemical analysis, estimates of the percentage of total epithelial cells in S, G2 and M were derived from the cyclin A LI, the cyclin B1 LI and the phosphohistone H3 LI, respectively. An estimate of the percentage of cells in G1 was derived by subtracting the combined LIs of cyclin A, cyclin B1 and phosphohistone H3 from the Mcm-2 LI. An estimate of the percentage of epithelial cells in G0 was derived from the percentage of total epithelial cells that were Mcm-2 negative.

### Analysis of borderline serous neoplasms

We investigated whether cell cycle parameters varied within the group of 21 borderline serous neoplasms. Information regarding tumour recurrence and outcome was not available for our sample group; therefore, direct comparisons between cell cycle phase distribution and the likelihood of recurrence could not be made. An estimate of malignant potential was made, therefore, based on histopathological features. The borderline tumours were graded on a scale from 1+ to 3+ for cytological atypia and architectural complexity by a specialist in gynaecological pathology (MJA). Grading was based on the following features: 1+, mild architectural complexity with some multilayering/stratification of cells with nuclei showing mild atypia; 2+, moderate architectural complexity with papillary projections, moderate mutilayering/stratification of cells with nuclei showing moderate nuclear atypia; and 3+, marked architectural complexity with papillary projections and papillary branching and marked nuclear atypia. These grades were then compared to the Mcm-2 LI, cyclin A LI and the cyclin A LF for each group.

### Statistical analysis

The LIs and LFs for the putative phase markers were compared using the Kruskal–Wallis test. Pairwise comparisons from these data were made by comparison of the median values (Mann–Whitney *U* test). The Wilcoxon signed rank test with exact *P*-values was used for paired comparisons between the percentages of cells in each phase of the cell cycle determined by flow cytometry and immunohistochemistry. Progressive differences for each putative cell cycle phase marker across the groups of neoplasms were compared using the Jonckheere–Terpstra (J-T) test, a nonparametric statistical test to detect a shift in ordered distributions when stratified by ordered categories.

## RESULTS

### Expression of MCM-2

**Figure 1:**
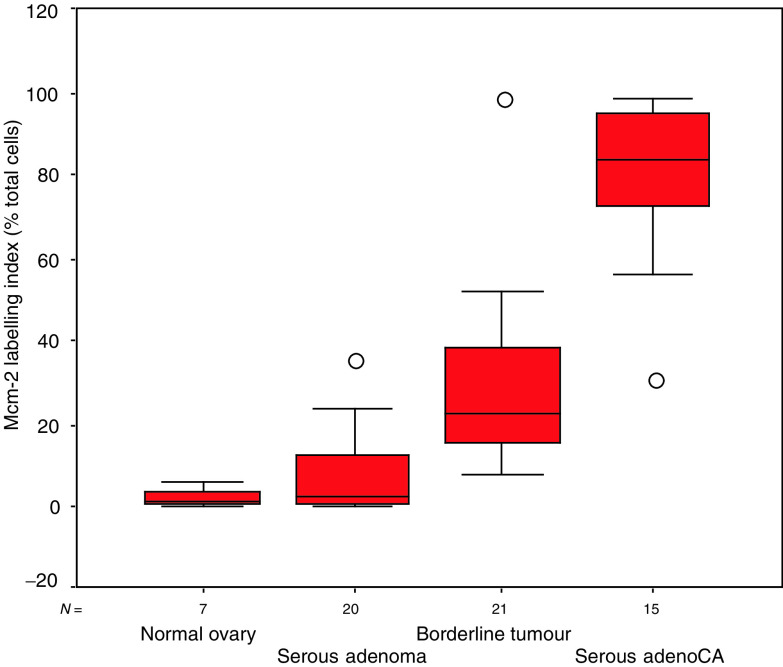
MCM-2 LIs in normal ovarian epithelium and serous ovarian neoplasms. Box and whisker plot comparing the Mcm-2 LI in normal ovary, serous cystadenomas, borderline serous tumours and serous cystadenocarcinomas. Line=median; box=IQR; whisker=2 × IQR; circles=extreme values.

**Figure 2:**
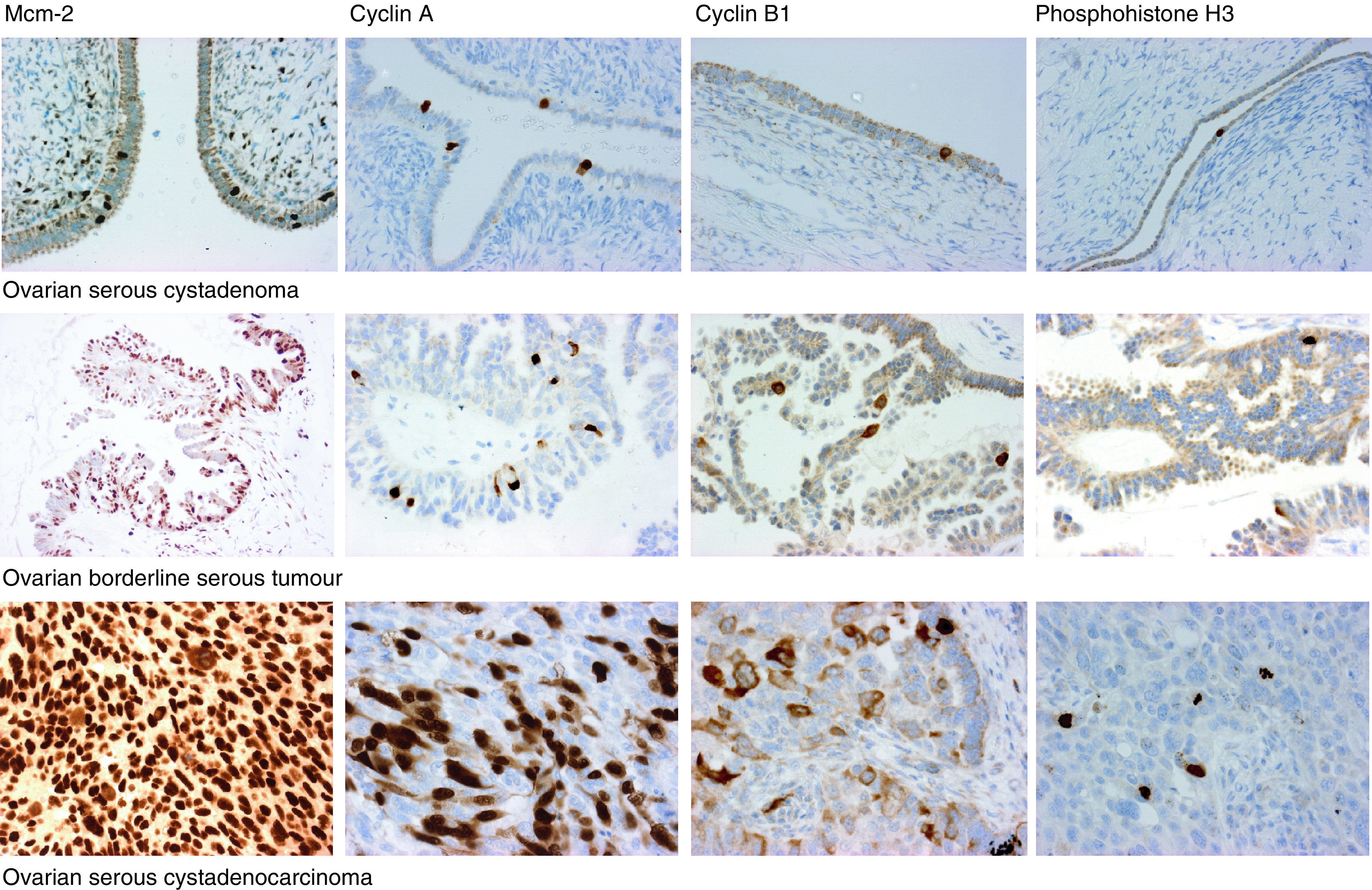
Immunoperoxidase staining for cell cycle markers in serous ovarian neoplasms. The figure illustrates the patterns of expression of Mcm-2 (1st column), cyclin A (2nd column), cyclin B1 (3rd column) and phosphohistone H3 (4th column) in serous cystadenomas (1st row), borderline serous tumours (2nd row) and serous cystadenocarcinomas (3rd row). Whereas in cystadenomas there is only an occasional Mcm-2 positive epithelial cell, in borderline tumours the epithelium shows widespread positivity for Mcm-2 and there is diffuse staining in the cystadenocarcinomas. Cyclin A and cyclin B1 LIs also increase with progression towards a malignant phenotype. Note the predominantly cytoplasmic staining for cyclin B1, consistent with cells in G2, but with occasional diffuse staining, consistent with entry into mitosis. Phosphohistone H3 detects only occasional nuclei in the cystadenomas and borderline tumours, whereas cystadenocarcinomas show numerous positive nuclei, including mitotic figures.

In the normal ovary, Mcm-2 staining was seen in the epithelial lining of small serous inclusion cysts. The number of cells expressing Mcm-2 was low, with a median LI of 1.4% (Figure 1

). In the serous cystadenomas, the cyst wall was attenuated and the epithelium formed a single layer of cuboidal or columnar cells. In these lesions, the median Mcm-2 LI was 2.7%. The borderline serous tumours showed a median Mcm-2 LI of 22.2%, with expression seen both in the lining of attenuated cyst walls and in papillary projections. The Mcm-2 LI was significantly in excess of that seen in the serous cystadenomas (*P*<0.0001). The median Mcm-2 LI for the serous cystadenocarcinomas was 80.4%. Expression was diffuse within the serous cystadenocarcinomas, although there was reduced expression in areas of necrosis. The Mcm-2 LI in serous cystadenocarcinomas was significantly in excess of that seen in borderline tumours (*P*<0.0001). There was a significant increase in Mcm-2 expression on progression from normal ovary through serous cystadenomas and borderline serous tumours to serous cystadenocarcinomas (*P*<0.0001; J-T test) (Figures 1 and 2

, first column).

### Expression of cell cycle phase markers

**Figure 3:**
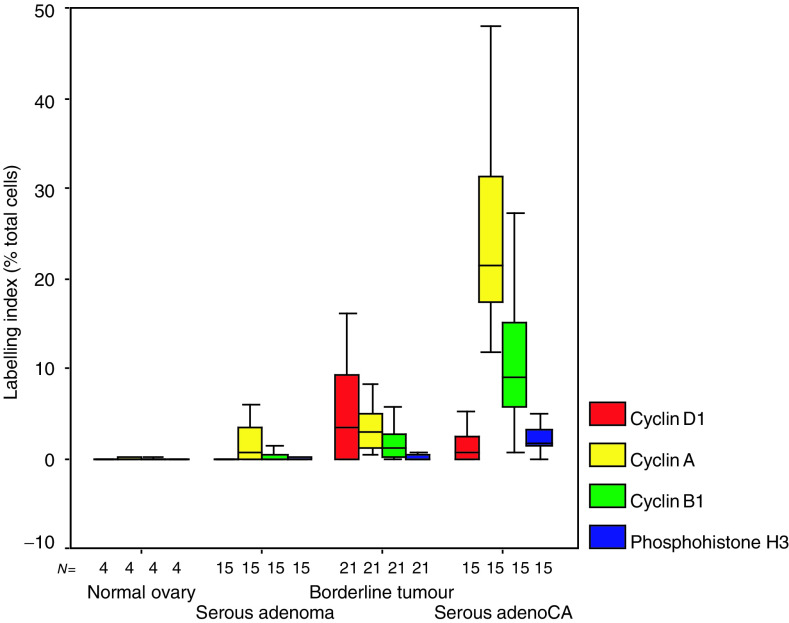
Cell cycle phase marker LIs in normal ovarian epithelium and serous ovarian neoplasms. Comparison of cell cycle phase marker expression (LIs) in normal ovary (normal ovary), serous cystadenomas (serous adenoma), serous borderline tumours (borderline tumours) and serous cystadenocarcinomas (serous adenoCA). Line=median; box=IQR; whisker=2 × IQR.

Cyclin D1 showed low-frequency nuclear expression in all sample groups. Occasional cells additionally showed cytoplasmic staining for cyclin D1, but these were not scored as cytoplasmic staining probably represents background or aberrant antigen retrieval. Scattered immunopositivity was seen in both borderline tumours and serous cystadenocarcinomas but there were no specific distribution patterns. Cyclin A showed a nuclear pattern of staining similar to that seen for Mcm-2, although, as expected, fewer cells were positive (Figure 2, second column). The pattern of staining with cyclin B1 was predominantly cytoplasmic (Figure 2, third column) with only occasional cells showing nuclear staining. Phosphohistone H3 showed both nuclear staining (presumed to be in prophase nuclei) and staining of mitotic figures (Figure 2, fourth column). There was a significant increase in the LIs for cyclin D1 (*P*=0.002; J-T test), cyclin A (*P*<0.0001; J-T test), cyclin B1 (*P*<0.0001; J-T test) and phosphohistone H3 (*P*<0.0001; J-T test) with progression from normal ovary through serous cystadenomas and borderline serous tumours to serous cystadenocarcinomas (Figure 3

). Although the LI for cyclin D1 appears to fall between borderline tumours and serous adenocarcinomas, this is not statistically significant (*P*=0.07) and the overall trend is towards an increase in expression by the Jonckheere–Terpstra test. Cyclin A and cyclin B1 LIs were elevated in borderline tumours compared to cystadenomas (*P*=0.003 and <0.0001, respectively) and in serous cystadenocarcinomas compared to borderline tumours (*P*=0.001 and <0.0001, respectively).

### Analysis of coexpression of cell cycle phase markers by double-labelling confocal microscopy

**Figure 4:**
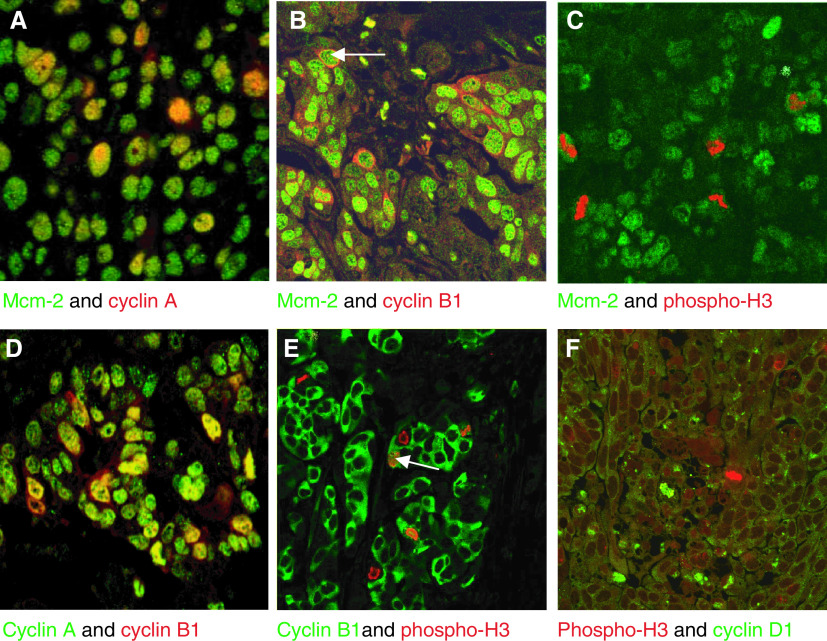
Double labelling confocal microscopy in serous cystadenocarcinomas. (**A**–**C**) Staining for Mcm-2 and phase-specific markers. (**A**) Mcm-2 (green) and cyclin A (red). The majority of cells express Mcm-2 (green). Occasional cells are yellow, representing cells positive for cyclin A in which Mcm-2 is coexpressed. No cells are positive for cyclin A in the absence of Mcm-2. (**B**) Mcm-2 (green) and cyclin B1 (red). Occasional Mcm-2 positive cells coexpress cyclin B1, which produces a red cytoplasmic halo in cells in G2 (arrow). (**C**) Mcm-2 (green) and phosphohistone H3 (red). Phospho-H3 stains the mitotic figures, in cells that have a green cytosol because of exclusion of Mcm-2 from condensed chromatin. (**D**–**F**) Staining for markers of adjacent cell cycle phases. (**D**) Cyclin A (green) and cyclin B1 (red) are coexpressed in a maximum of 5% of cyclin A-positive cells. (**E**) Cyclin B1 (green) and phosphohistone H3 (red) are coexpressed in less than 2% of phosphohistone H3-positive cells, unless the cyclin B1 staining is nuclear, indicating entry into prophase of mitosis (arrow). (**F**) There is no coexpression of phosphohistone H3 (red) and cyclin D1 (green).

There was coexpression of Mcm-2 in all epithelial cells from cystadenocarcinomas and serous borderline tumours that expressed one of the cell cycle phase markers cyclins D1, A, B1 and phosphohistone H3 (Figure 4a–c

). We also performed double staining to assess the degree of coexpression of adjacent cell cycle phase markers in serous cystadenocarcinomas and borderline serous tumours (Figure 4d–f). There was no coexpression of phosphohistone H3 and cyclin D1 in any lesion examined, consistent with the notion that cyclin D1 is detectable *in vivo* during its phase of maximal expression in mid-to-late G1, but not in early G1. There were occasional cells showing coexpression of cyclin D1 with cyclin A (<5% of cyclin A-positive cells), presumably identifying those cells around the G1/S transition. There were very infrequent neoplastic cells showing coexpression of cyclin A and cyclin B1 (5% of cyclin A-positive cells), suggesting that cyclin A is detectable *in vivo* during its phase of maximal expression in S, but not substantially in G2, similar to the staining pattern that we have observed in the colon (Scott *et al*, 2003). In the cystadenocarcinomas, there was occasional coexpression of cytoplasmic cyclin B1 with nuclear phosphohistone H3 (approx 1% of phosphohistone H3-positive cells), most likely representing cells around the G2/M transition.

### Phase distribution of cycling cells

**Figure 5:**
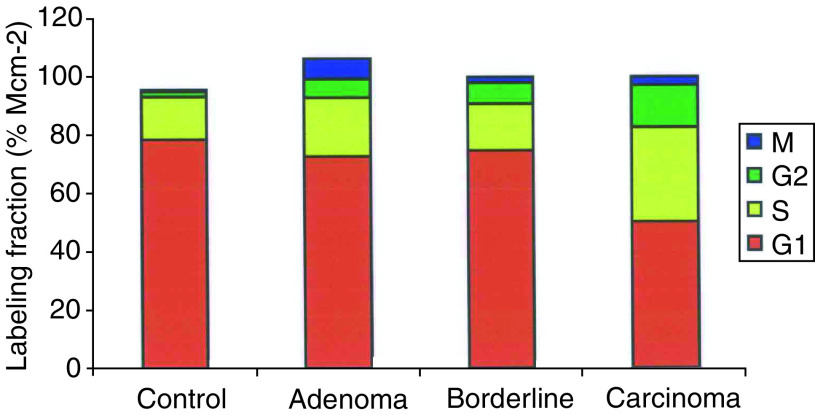
Cell cycle phase distribution in cycling cells. Stacked bar graph illustrating the phase distribution of cells in each sample group determined as fractions of the number of cells in cycle (as defined by the expression of Mcm-2). Control=normal ovary; adenoma=serous cystadenoma; borderline=borderline serous tumour; carcinoma=serous cystadenocarcinoma. S phase is determined from the cyclin A LF, G2 from the cyclin B1 LF and M from the phosphohistone H3 LF. G1 is the difference between the Mcm-2 LI and the sum of each of the above LFs. The S and G2 fractions increase across the groups (*P*=0.003 and <0.0001, respectively), and there is a corresponding decrease in the G1 fraction (*P*=0.0008).

The frequency of expression of each phase marker (LI) was expressed as a percentage of the Mcm-2 positive cells to produce a labelling fraction (LF) (Figure 5

). The Mcm2-positive, phase marker-negative cells were assumed to be in early G1 and these cells were included with the cyclin D1-positive cells when determining the G1 fraction. Application of the Jonckheere–Terpstra test to these phase distribution results showed a decrease in G1 fractions (*P*=0.0008), and an increase in cyclin A (S phase; *P*=0.003) and cyclin B1 (G2 phase; *P*<0.0001) LFs on progressing through the spectrum of serous neoplasia. There was no significant change in the LF for phosphoshistone H3 (mitosis). These data are consistent with increased S phase entry in cells of cystadenocarcinomas compared to cells of borderline tumours and cystadenomas.

### Comparison of cell cycle phase distributions determined by flow cytometry and immunohistochemistry

In total, 10 representative cases of serous cystadenocarcinoma were examined by flow cytometry in addition to immunohistochemistry. There was no significant difference in the S-phase fractions and G2/M-phase fractions when estimated by the two methods (*P*=0.17 and 0.06, respectively). In contrast, there was a difference in the G0/G1 fractions determined by the two methods (*P*=0.017), with flow cytometric analysis giving a lower estimate of the number of cells in G0/G1 presumably due to nuclear fragmentation.

### Borderline serous tumours: associations between grade and cell cycle parameters

**Figure 6:**
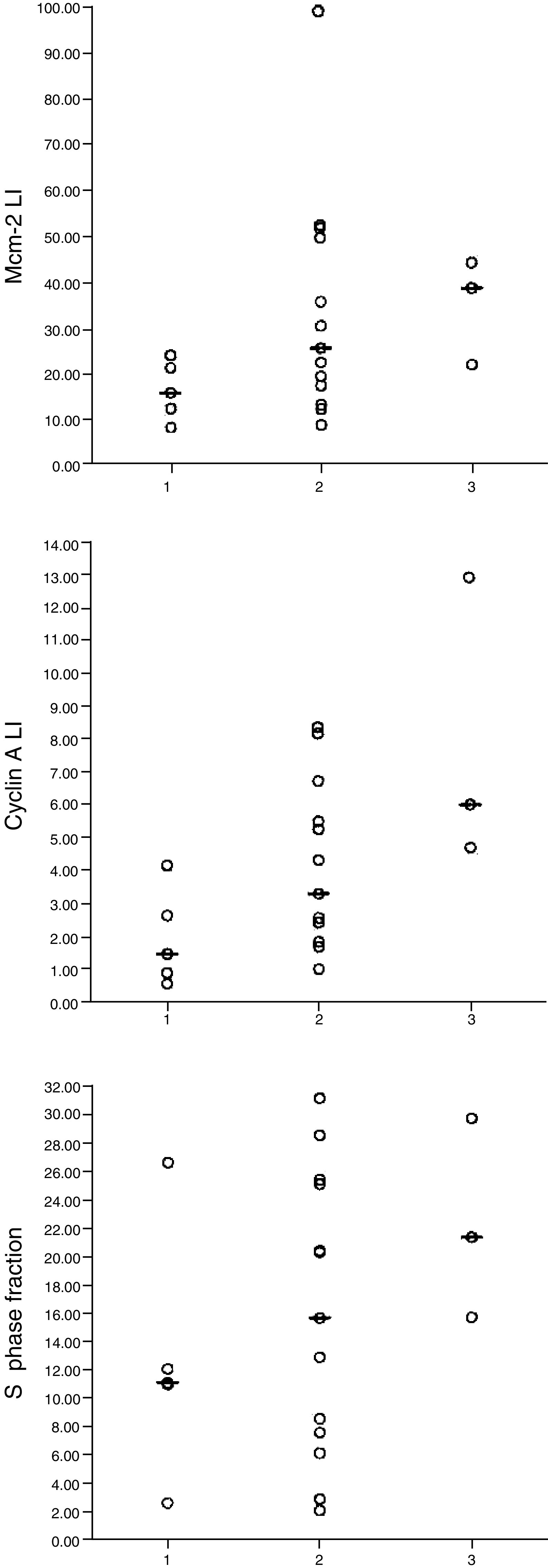
Cell cycle parameters in borderline serous tumours of different grades. Values obtained for Mcm-2 LI (top), cyclin A LI (middle) and the S-phase fraction (cyclin A LF, bottom) in borderline serous tumours scoring 1+ (left column), 2+ (middle column) and 3+ (right column) for both architectural and cytological atypia.

The 1+ borderline tumours had a lower Mcm-2 LI than either the 2+ or 3+ tumours (*P*=0.04), but there was no significant difference between the 2+ and 3+ tumours. There was a stepwise increase in both cyclin A LI and the S-phase fraction (cyclin A LF) as the histological features increased from 1+ through 2+ to 3+. This suggests that either the cyclin A LI or the S-phase fraction has the potential to aid discrimination between different grades within this group of serous borderline tumours (Figure 6

).

## DISCUSSION

Ovarian serous tumours of borderline malignant potential will normally behave in an entirely benign manner; however, 15–30% of these tumours have the potential for aggressive behaviour and may exhibit malignant change. This hypothesis is based on ovarian tumours showing a continuum of progression from serous borderline tumours to serous cystadenocarcinomas. This concept is widely accepted and is based largely on both histopathological observation of foci of microinvasion within borderline tumours and cytogenetic evidence showing similar chromosomal alterations in both types of ovarian neoplasm, and also in serous adenomas, particularly those involving chromosome 6 (Tibiletti *et al*, 2001, 2003). There is evidence, however, to suggest that each of these tumour entities may arise *de novo* and these studies are based largely on the patterns of mutations in *TP53* and *K-RAS*, each of which can show considerable heterogeneity (Teneriello *et al*, 1993; Herbst, 1994; Miki *et al*, 1994; Ortiz *et al*, 2001). A further model suggests a dual pathway in which some, often the more aggressive tumours, arise *de novo* and others, the majority, arise by stepwise progression from borderline tumours (Singer *et al*, 2002, 2003). Our data, showing a consistent progression of markers of cell cycle entry and cell cycle phase, would favour the stepwise progression model in this small subset of serous neoplasms.

Currently, a diagnosis of borderline tumour is made where there are histologically atypical features without evidence of destructive stromal invasion. No specific marker has yet been identified, however, that is able to separate reliably this diagnostic entity into benign and malignant prognostic groups. An accurate indication of cell cycle state and phase distribution may be of value in this regard.

An immunohistochemical method to estimate cell cycle phase distribution *in situ* using tissue sections has considerable advantages in the routine diagnostic setting compared to existing methods of cell cycle analysis such as flow cytometry (Scott *et al*, 2003) and overall comparisons suggest that our immunohistochemical method is able to produce cell cycle analyses comparable to those obtained by flow cytometry.

A potential criticism of an immunohistochemical method, such as the one described here, is that the counts may not be reproducible as only a small proportion of the total cells are assessed. In our study the cell cycle counting was performed by three individuals. The interobserver variation of less than 5% that we observed is comparable with values from many other methods and offers the advantage that areas of tumour heterogeneity can be identified and morphologically different areas can be assessed.

We have observed in a parallel study that Mcm-2 was expressed in more cells in normal and neoplastic ovary than the traditional marker Ki67 (data not shown). Moreover, antibodies against Ki67 fail to stain all the cells labelling with phosphohistone H3 and occasional cells labelling with cyclin D1 (Chatrath *et al*, 2003). In contrast, Mcm-2 identifies every cell expressing a marker of cell cycle phase, demonstrating its value as a marker of cell cycle entry (Scott *et al*, 2003). The Mcm-2 positive, cell cycle phase marker negative cells were assumed to be in early G1 but, as there is no reliable marker of this phase of the cell cycle, this hypothesis cannot be substantiated at the current time. Nevertheless, our data suggest that Mcm-2 may offer advantages over Ki67 as a marker of the cell cycle ‘state’ of tissues.

Confocal microscopy suggested that cyclins D1, A and B1 and phosphohistone H3 do not show any significant coexpression in ovarian neoplasms, unlike data from some tumour cell lines (Pines and Hunter, 1992). The relative S-phase specificity of immunodetectable cyclin A in ovarian tumours was demonstrated by the minimal coexpression of cyclin A with cyclins D1 and B1, used as markers of mid-to-late G1 and G2 phases, respectively, and the good correlation with flow cytometric estimates of S-phase cells. Similar observations were also made in our earlier study of colorectal carcinoma (Scott *et al*, 2003), in which cyclin A expression was also not detected in any cell not seen to be actively replicating DNA using our *in situ* replication assay (Mills *et al*, 2000). In order for *in situ* DNA replication to be effective, the tissue must be rapidly removed and snap-frozen in liquid nitrogen to preserve the DNA synthetic machinery within the cells. Such a study could not be repeated for the ovarian neoplasms because of the prolonged ischaemic time involved in gynaecological surgery. Taken together, our data suggest that cyclin A expression can be used as a surrogate immunohistochemical marker of S phase and that the cyclin A/Mcm2 ratio in ovarian neoplasms can be used to estimate the fraction of cycling cells that are in the S phase.

We observed an increase in the LIs of Mcm-2, cyclins D1, A and B1 and phosphohistone H3 in ovarian serous cystadenocarcinomas compared to borderline serous tumours, serous cystadenomas and normal ovary. The findings we describe for Mcm-2 are similar to those obtained in previous studies using Ki67 (Garzetti *et al*, 1995; Blegen *et al*, 2000). Indeed, it has been shown previously that tumour cell proliferation, as assessed by the Ki67 LI, was significantly higher in cases of serous ovarian cystadenocarcinoma that recurred or progressed than in those that did not (Garzetti *et al*, 1995). In the present study, we did not observe the very high frequency of expression of MCMs that was seen in serous inclusions in four cases of normal ovary in a previous study (Freeman *et al*, 1999).

Increases in the cyclin D1 LIs were observed in the serous cystadenocarcinomas. Dhar *et al* (1999) found 13.5% of serous ovarian cystadenocarcinoma cells to be cyclin D1 positive compared with 1.58% in our study. This disparity is most likely to be due to differences in antigen retrieval. We have found anti-cyclin D1 antibodies difficult to work with. Variations in the time and method of antigen retrieval can alter the binding characteristics and cellular localisation of this antibody by a considerable margin (±20%), while still producing minimal background staining. The apparent reduction in cyclin D1 expression on progression from borderline tumours to serous cystadenocarcinomas may reflect dysregulation of the G1/S checkpoint in the higher grade lesions as cyclin D1 is known to accumulate in the presence of an intact G1/S checkpoint (Berardi *et al*, 2003).

Determination of cyclin A LFs may be of greater value in predicting outcome and/or the likely response to chemotherapy, by indicating the fraction of cycling tumour cells (rather than the total number of tumour cells) that are in S phase. Indeed, we have shown previously in colorectal carcinomas that cyclin A LFs give a greater range of values for S-phase cells than do cyclin A LIs or flow cytometry (Scott *et al*, 2003). When investigating whether cell cycle parameters varied within the group of borderline serous neoplasms, we observed an increase in Mcm-2 LI, cyclin A LI and the cyclin A LF (S-phase fraction) with increasing histopathological grade. It may be that these parameters would be of clinical value in predicting outcome, as has been shown in other types of malignancy, such as breast carcinoma (Gonzales *et al*, 2003). Testing this hypothesis would require a much larger series of cases for which there was adequate outcome data, taking into account histological grade and stage, treatment received and with corrections for lead and lag-time bias.

In conclusion, the immunohistochemical method for cell cycle assessment that we describe offers numerous practical benefits, in that it is reproducible, can be standardised in any diagnostic histopathology laboratory and the analysis can be confined to the neoplastic component. Unlike flow cytometric assessment of homogenised samples, the method enables separate examination of multiple sites within a tumour, allowing an evaluation of the considerable heterogeneity that can exist. We have demonstrated that in ovarian serous neoplasms, expression of all markers of cell cycle state and phase increased on progression from benign through borderline to malignant tumours. Estimation of parameters such as Mcm-2 LI or cyclin A LF (S-phase fraction) in tissue sections may prove to be a highly convenient means of predicting clinical outcome in borderline serous tumours and serous cystadenocarcinomas.
